# Ear, Nose, and Throat Manifestations in Inflammatory Bowel Diseases: A Systematic Review of the Clinical Spectrum

**DOI:** 10.3390/medicina62050943

**Published:** 2026-05-12

**Authors:** Eleni Litsou, Georgios Psychogios, Maria Saridi, Konstantinos H. Katsanos, Fotios Fousekis

**Affiliations:** 1 Department of Otorhinolaryngology, Head and Neck Surgery, University Hospital of Ioannina, 45500 Ioannina, Greece; 2 Department of Nursing, University of Thessaly, 41500 Larissa, Greece; 3 Department of Gastroenterology, University Hospital of Ioannina, 45500 Ioannina, Greece

**Keywords:** inflammatory bowel disease, ear, nose and throat manifestations, sensorineural hearing loss, nasal pyoderma gangrenosum, laryngeal involvement

## Abstract

*Background*: Inflammatory bowel disease (IBD), including ulcerative colitis (UC) and Crohn’s disease (CD), represents a chronic immune-mediated disorder frequently associated with extraintestinal manifestations. While musculoskeletal, dermatologic, and ocular complications are well recognized, ear, nose, and throat (ENT) involvement remains underrecognized despite its potential morbidity. *Objective*: To systematically evaluate the spectrum of ENT manifestations in IBD, focusing on clinical presentation, diagnostic approaches, and outcomes. *Methods*: A systematic literature search was conducted in PubMed and Scopus in accordance with PRISMA 2020 guidelines. Eligible studies included English-language human studies (2015–2026) reporting ENT manifestations in UC or CD. Following screening, 23 studies were included in the qualitative synthesis. Extracted data comprised study design, IBD subtype, patient demographics, ENT manifestations, diagnostic methods, and clinical outcomes. *Results*: The majority of studies consisted of case reports and small observational series. Sensorineural hearing loss (SNHL) was the most frequently reported manifestation in both adult and pediatric populations, with evidence suggesting immune-mediated mechanisms and variable responsiveness to corticosteroids. Nasal involvement included pyoderma gangrenosum, pyoderma vegetans, and aseptic nasal septal abscess, occasionally resulting in severe structural complications such as saddle-nose deformity. Laryngeal and airway involvement included dysphonia, tracheitis, and rare but potentially life-threatening inflammatory airway disease. Additional findings included associations with chronic rhinosinusitis. Diagnosis relied on audiometry, imaging, endoscopy, and histopathology. Systemic corticosteroids were frequently effective; however, delayed recognition may lead to irreversible sequelae. *Conclusions*: ENT manifestations in IBD constitute a clinically heterogeneous but important group of extraintestinal complications. Increased awareness of ENT manifestations may support earlier diagnosis and multidisciplinary management of IBD, potentially reducing irreversible complications.

## 1. Introduction

Inflammatory bowel disease (IBD) represents a chronic, relapsing inflammatory disorder of the gastrointestinal tract that arises from dysregulated immune responses to intestinal microbiota in genetically predisposed individuals [1]. The two principal subtypes, ulcerative colitis (UC) and Crohn’s disease (CD), differ in anatomical distribution and depth of intestinal involvement. UC typically presents as a continuous inflammatory process confined to the colonic mucosa, most commonly beginning in the rectum and extending proximally to involve part or the entire colon [2]. In contrast, CD is characterized by transmural inflammation, discontinuous “skip” lesions, and the potential to affect any segment of the gastrointestinal tract, most frequently the terminal ileum and colon [2,3]. CD may manifest with inflammatory, stricturing, or penetrating phenotypes, contributing to complications such as fistula formation and bowel obstruction [2].

IBD has been associated with substantial morbidity, impaired quality of life, and long-term complications [4]. Diagnosis is typically based on clinical features, cross-sectional imaging, and endoscopic evaluation with histopathological evaluation [5]. Beyond intestinal inflammation, both UC and CD frequently present with extraintestinal manifestations, reflecting systemic immune dysregulation and inflammatory activity beyond the gastrointestinal tract [2,6]. The most frequently affected extraintestinal sites include the musculoskeletal system, skin, and ocular tissues, and these manifestations may occur independently of intestinal disease activity [2,5]. Moreover, chronic inflammation in IBD increases the risk of long-term complications, including colorectal malignancy, particularly in patients with long-standing extensive colitis [7,8].

Although extraintestinal involvement is well recognized, manifestations affecting the ear, nose, and throat (ENT) remain less systematically described in the literature. ENT symptoms in IBD may reflect inflammatory involvement of the upper aerodigestive tract, immune-mediated mucosal disease, secondary infections related to immunosuppressive therapy, or complications associated with systemic inflammation. Such manifestations may be overlooked or misattributed to infectious or allergic conditions, potentially delaying appropriate diagnosis and management. To our knowledge, a focused synthesis of ENT manifestations in IBD across otologic, sinonasal, and airway domains remains limited. Therefore, the present systematic review aimed to systematically evaluate the spectrum of ENT manifestations in patients with IBD, including CD and UC, with particular emphasis on clinical presentation, diagnostic approaches, and reported outcomes.

## 2. Materials and Methods

### 2.1. Protocol and Reporting Standards

This review followed PRISMA 2020 recommendations. The review protocol was not prospectively registered; however, the methodology and eligibility criteria were defined prior to study screening to minimize bias. PRISMA provides a 27-item checklist to ensure transparent, complete, and reproducible reporting of systematic reviews and meta-analyses, including detailed documentation of search strategies, study selection, and data extraction [9]. The PRISMA flow diagram was used to illustrate the identification, screening, eligibility, and final inclusion of studies.

### 2.2. Eligibility Criteria

Studies were eligible for inclusion if they met the following criteria:

- Original research studies (including case reports, case series, observational studies, and clinical investigations) reporting ENT manifestations in individuals with clinically diagnosed IBD (CD or UC).
- Published in English language.
- Published from 1 January 2015 onward to capture contemporary clinical evidence and relevance.
- Human studies.

Studies were excluded if they were reviews, editorials, letters without primary data, conference abstracts only, animal studies, or did not report ENT outcomes specific to IBD.

Eligibility criteria were defined before study selection to minimize bias and enhance reproducibility. Systematic reviews rely on explicit eligibility criteria to ensure that only studies directly relevant to the research question are included.

### 2.3. Information Sources and Search Strategy

A systematic literature search was conducted in PubMed and Scopus, selected for their comprehensive biomedical coverage and inclusion of case reports and observational studies. Searches were performed on 25 January 2026 and were limited to English-language publications. The search strategy combined MeSH terms and relevant keywords. Search terms included “inflammatory bowel disease”, “ulcerative colitis”, and “Crohn’s disease”, combined with terms related to ear, nose, and throat (ENT) involvement, including “ear”, “nose”, “throat”, “ENT”, “hearing loss”, “sensorineural hearing loss”, “sinusitis”, “rhinosinusitis”, “nasal”, “laryngeal”, “airway”, and “voice”. Boolean operators (AND, OR) were applied to structure the search appropriately. All search results were documented and exported in standard bibliographic formats (e.g., .nbib from PubMed and .ris from Scopus) for subsequent screening and deduplication. The full search strategy for each database is provided in the Supplementary Material.

### 2.4. Study Selection and Data Management

All retrieved records were imported into a reference management system, where duplicates were identified and removed. Title and abstract screening were performed independently to assess relevance, and decisions were recorded systematically. Full-text assessment was then conducted to confirm eligibility.

**Figure 1:**
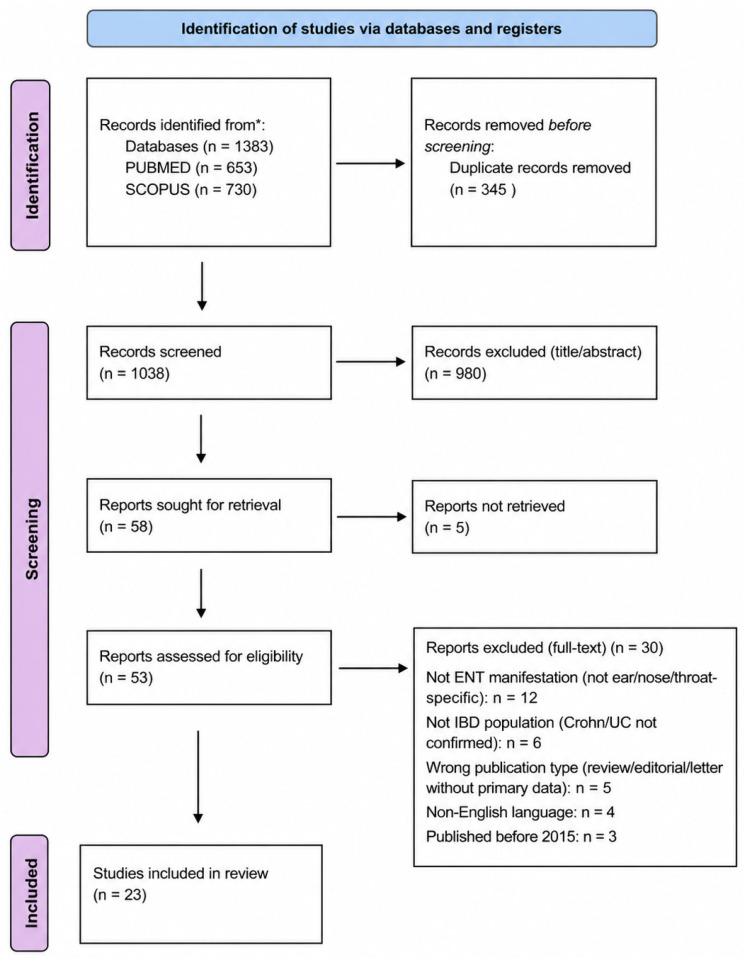
PRISMA 2020 flow diagram of study selection. A total of 1383 records were identified through database searching (PubMed *n* = 653; Scopus *n* = 730). After removal of 345 duplicates, 1038 records were screened by title and abstract. Of these, 985 were excluded. Fifty-three full-text articles were assessed for eligibility, and 30 were excluded for various reasons. Ultimately, 23 studies were included in the qualitative synthesis. * Indicates statistical significance. The arrows indicate the direction/relationship between the different components, while the different colors are used to distinguish the individual categories/groups presented in the figure.

The PRISMA flow diagram (Figure 1) illustrates the study selection process, including the number of records identified, screened, excluded (with reasons), and included for qualitative synthesis. This ensures transparency and adherence to PRISMA recommendations [9].

### 2.5. Data Extraction

For each study included after full-text review, the following information was systematically extracted:

- Study characteristics: study design, setting, sample size.
- Population details: IBD subtype (UC or CD), age, sex.
- ENT manifestations: specific symptom(s) or diagnosis reported.
- Diagnostic methods: criteria, investigations, or procedures used to confirm ENT involvement.
- Clinical outcomes: key findings, response to therapy, and disease course.

Data extraction was piloted on a subset of studies to ensure consistency and reliability. Extracted information was recorded in a structured spreadsheet, enabling comparison across studies and identification of patterns in ENT manifestations related to IBD. 

### 2.6. Quality Assessment

Due to the predominance of case reports and small observational studies among the included publications, a formal quantitative risk-of-bias assessment was not considered appropriate. Nevertheless, methodological quality was evaluated qualitatively based on study design, diagnostic confirmation of ENT manifestations, and clarity of clinical outcome reporting.

## 3. Results

The study selection process is summarized in Figure 1. A total of 1383 records were identified through database searching. After removal of duplicates (*n* = 345), 1038 records were screened, and 23 studies were included in the qualitative synthesis.

This structured approach allowed comparison of ENT manifestations across IBD subtypes and facilitated the identification of the most frequently affected sites, including ear, nose, larynx, and airway, and evaluation of diagnostic approaches and treatment responses. Patterns in audiovestibular, nasal, laryngeal, and airway involvement were noted, highlighting the clinical diversity of ENT extraintestinal manifestations in IBD.

### 3.1. Study Characteristics

A total of 23 studies met the inclusion criteria, predominantly consisting of case reports, case series, and small observational studies, including case–control and prospective designs. This distribution reflects the relative rarity and under-recognition of ENT manifestations in IBD. Larger-scale evidence was limited, with only a small number of observational and prospective investigations available.

The most frequently reported ENT manifestations were:

1. Audiological involvement, particularly sensorineural hearing loss (SNHL).
2. Nasal inflammatory lesions, including destructive processes.
3. Upper airway and laryngeal involvement.

### 3.2. Ear Manifestations

#### Sensorineural Hearing Loss

The most frequently reported ENT manifestation across the included studies was SNHL. A retrospective study identified 32 IBD patients with hearing loss over 17 years [10]. Most cases developed after IBD onset, with a median interval of 9 years; 63% occurred within the first decade. Audiometric patterns were heterogeneous, ranging from mild to profound. Sudden-onset unilateral hearing loss and vestibular symptoms (vertigo, tinnitus) were frequent.

Early high-dose corticosteroid therapy showed potential benefit in selected patients, whereas chronic low-dose steroids did not prevent progression. Pediatric evidence [11] has also suggested the presence of subclinical high-frequency SNHL, supporting the possibility of early cochlear involvement. Observational evidence has also described patterns of hearing impairment among patients with IBD [12]. However, other studies found no significant differences in SNHL among UC patients in remission versus controls, suggesting that SNHL may affect only a subset of IBD patients, potentially influenced by immune-mediated mechanisms or disease activity [13].

Case reports further support SNHL as a potential extraintestinal manifestation of IBD. Several reports have described immune-mediated presentations, sometimes accompanied by neurological complications. Tirelli et al. [14] described sudden SNHL in a patient with CD, raising suspicion for autoimmune inner ear involvement. Similarly, Vahedi et al. [15] and Yazici et al. [16] have documented SNHL occurring alongside severe neurological manifestations in newly diagnosed UC patients. Additionally, Birda et al. [17] reported SNHL in a patient with UC, further highlighting the potential link between immune-mediated inner ear dysfunction and intestinal inflammatory activity.

### 3.3. Audiovestibular Autoimmune Syndromes

Cogan’s syndrome represents a rare audiovestibular autoimmune condition reported in association with inflammatory bowel disease. A case series of 22 patients (6 with UC and 16 with CD) demonstrated that approximately half of the cases occurred during periods of active intestinal disease [18]. Notably, ongoing immunomodulatory therapy, including anti-TNF (tumor necrosis factor) agents, did not appear to prevent syndrome development. Clinical responses to corticosteroid-based treatment were often partial, supporting an immune-mediated pathogenesis that may occur independently of intestinal disease activity.

### 3.4. Nose Manifestations

#### Nasal Pyoderma Gangrenosum and Destructive Lesions

Nasal inflammatory lesions were rare but a clinically significant manifestation in patients with IBD. Reported cases included UC-associated pyoderma gangrenosum mimicking a septal abscess and progressing to saddle-nose deformity despite corticosteroid response [19]. Other reports described nasal mucosa pyoderma vegetans associated with UC responsive to systemic corticosteroid therapy [15,20], as well as aseptic septal abscess in PR3-ANCA-positive UC, treated successfully with glucocorticoids and rituximab [21]. These observations highlight the importance of considering systemic inflammatory disease, including IBD, in patients presenting with severe or atypical nasal lesions. Rare presentations of CD involving the nasal cavity have also been reported. Zaseeva et al. [22] described CD presenting as an intranasal space-occupying lesion, highlighting that granulomatous inflammation may mimic neoplastic or infectious processes in the nasal cavity.

### 3.5. Subclinical Nasal Inflammation

Evidence also suggests the presence of subclinical sinonasal involvement in patients with IBD. Increased lymphocytes in nasal cytology have been demonstrated to correlate with the grade of intestinal inflammation, suggesting subclinical sinonasal mucosa activity [23]. In addition, chronic rhinosinusitis (CRS) has been reported in patients with suspected or confirmed IBD, highlighting a bidirectional association between gut and sinonasal inflammation [24]. Inflammatory conditions affecting the nasal cavity and adjacent structures may share common mucosal inflammatory pathways with other craniofacial inflammatory disorders [25].

### 3.6. Laryngeal and Airway Manifestations

#### Upper Airway Inflammation

Severe pediatric airway involvement was reported in two UC cases presenting with tracheitis [26], both of whom improved rapidly following systemic corticosteroid therapy. Colevas et al. [27] described granulomatous supraglottic edema requiring intensive airway management, highlighting the potentially life-threatening nature of airway manifestations in IBD. Rare inflammatory lesions of the supraglottic region have also been described in CD. Hori et al. [28] reported refractory aphthous lesions involving the epiglottis and supraglottic region, further supporting that inflammatory involvement of upper airway mucosa may occur in patients with IBD. Additional laryngeal and airway involvement has also been reported in CD. Yin et al. [29] described inflammatory laryngeal lesions presenting with dysphonia, while Nakai et al. [30] reported tracheobronchitis and laryngitis associated with CD, suggesting that IBD may affect both upper and lower airway mucosa beyond the gastrointestinal tract.

### 3.7. Tonsillar Disease and Infectious Complications

Infectious ENT complications may also occur in patients with IBD, particularly in the context of systemic inflammation or immunosuppressive therapy. Severe complications such as Lemierre syndrome, originating from oropharyngeal infection and leading to internal jugular vein thrombosis, have been described in adolescents with active UC [31]. In addition, immunosuppressive therapy used in IBD management may predispose patients to opportunistic infections affecting the oral and pharyngeal mucosa [32]. Large population-based studies have also demonstrated an increased risk of opportunistic infections among patients receiving immunosuppressive treatment for IBD [33].

### 3.8. Voice Impairment

Voice-related manifestations have also been reported in patients with inflammatory bowel disease. Inflammatory involvement of the laryngeal mucosa may lead to symptoms such as dysphonia or voice changes, reflecting laryngeal participation in the spectrum of extraintestinal manifestations [34].

### 3.9. Malignancy-Related ENT Outcomes

#### 3.9.1. Laryngeal Cancer

Van de Ven et al. [35] reported laryngeal carcinoma risk factors in IBD: male sex, older age at UC diagnosis, and tobacco use or complicated phenotypes in CD. Survival was comparable to that of the general population, and immunosuppressive therapy did not affect outcomes.

#### 3.9.2. Thrombotic, Infectious, and Medication-Related ENT Complications

Rare ENT complications have also been reported in association with inflammatory bowel disease, including Lemierre syndrome, a septic thrombophlebitis of the internal jugular vein originating from oropharyngeal infection [31], as well as opportunistic infections such as mucormycosis in immunosuppressed patients with UC [30,36]. In addition, medication-related adverse events should also be considered in the differential diagnosis of ENT manifestations in patients with IBD. These findings also highlight the importance of distinguishing primary disease-related manifestations from medication-induced adverse effects, particularly in patients receiving immunosuppressive or anti-inflammatory therapy.

### 3.10. Summary of Diagnostic Approaches Across Studies

Diagnostic strategies varied significantly between studies. Hearing loss studies relied primarily on pure tone audiometry, high-frequency audiometry, and otoacoustic emissions testing. Nasal manifestations were commonly assessed using computed tomography imaging, endoscopic ENT examination, and histopathological confirmation when lesions were destructive or atypical. Laryngeal and airway manifestations were typically evaluated through Laryngofibroscopy and clinical progression. Large-scale epidemiological associations were derived from registry or database-based diagnostic coding and pathology confirmation.

### 3.11. Treatment Response and Clinical Outcomes

Across studies, systemic corticosteroids were frequently reported as beneficial in immune-mediated ENT manifestations, including hearing loss, nasal pyoderma gangrenosum, and airway inflammation. However, several reports emphasized that delayed recognition may lead to irreversible complications, such as saddle-nose deformity following nasal pyoderma gangrenosum.

In audiological disease, early high-dose steroid therapy appeared to be associated with improved outcomes in selected cases, whereas chronic low-dose steroid exposure did not reliably prevent progression of hearing loss.

**Figure 2:**
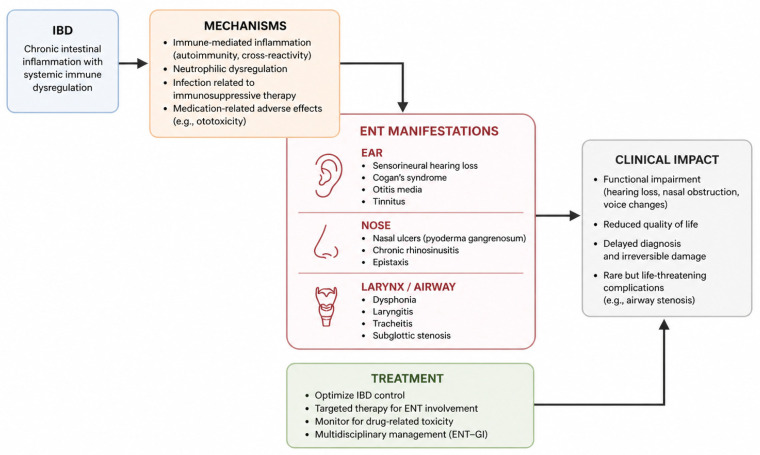
Conceptual framework of ear, nose, and throat (ENT) manifestations in inflammatory bowel disease (IBD). Systemic immune dysregulation in IBD contributes to ENT involvement through multiple mechanisms, including immune-mediated inflammation, neutrophilic processes, infection, and medication-related adverse effects. These mechanisms result in a broad spectrum of ENT manifestations affecting the ear, nose, and larynx/airway, with potential clinical consequences such as functional impairment, reduced quality of life, and irreversible structural damage. Treatment strategies and early recognition may improve outcomes.

The spectrum of ENT manifestations in IBD, including underlying mechanisms, clinical impact, and treatment considerations, is summarized in Figure 2.

## 4. Discussion

This review summarizes the available evidence on ENT manifestations associated with IBD, encompassing both UC and CD. Across the 23 included studies, ENT manifestations were identified as either (1) immune-mediated extraintestinal manifestations, (2) secondary complications of systemic inflammation, (3) infection-related complications linked to immunosuppressive therapy, or (4) drug-related adverse reactions affecting ENT structures. Extraintestinal manifestations occur in a significant proportion of patients with IBD and are believed to reflect systemic immune dysregulation affecting multiple organ systems [37,38]. ENT involvement in IBD appears heterogeneous and is often underrecognized, yet clinically important because of the potential for significant functional impairment (e.g., hearing loss or airway compromise) and irreversible structural damage (e.g., saddle-nose deformity).

An important contribution of the present review is the demonstration that ENT manifestations may affect multiple anatomical regions, including the ear, nasal cavity and paranasal sinuses, larynx, airway, and adjacent mucosal structures. Extraintestinal manifestations of IBD may involve multiple organ systems, including the skin, joints, eyes, and, less commonly, the head and neck region [39,40,41]. Previous literature has similarly described a wide spectrum of head and neck involvement associated with IBD, including otologic, laryngeal, and oral involvement [32,34,42,43,44]. IBD may also manifest in the oral cavity and upper aerodigestive mucosa, highlighting the broad spectrum of mucosal involvement in systemic inflammatory disease [45]. Oral and mucosal manifestations are also well recognized in IBD and may precede gastrointestinal symptoms in some patients [46]. These findings suggest that ENT symptoms in IBD should not be regarded as isolated abnormalities but rather as clinically relevant manifestations that may require multidisciplinary evaluation and management.

### 4.1. Overview of Evidence Quality and Study Characteristics

The evidence base identified in this review consisted predominantly of case reports and small observational studies, with only a limited number of larger cohort or database-driven investigations. This likely reflects the relative rarity and under- recognition of ENT manifestations in IBD, which are rarely captured as primary outcomes in gastroenterology research. In contrast, the otolaryngology literature more frequently reports such associations based on individual clinical observations. Consequently, robust epidemiological data regarding the true incidence and prevalence of ENT involvement in IBD remain limited.

Despite these limitations, several studies have attempted broader epidemiological evaluation. For example, a pathology-based case–control investigation explored risk factors and clinical outcomes of laryngeal carcinoma among patients with IBD [35]. Other studies examined inflammatory manifestations involving the upper airway or head and neck region in association with IBD, including [34]. Refractory aphthous lesions involve the epiglottis and supraglottic region in CD, illustrating that inflammatory involvement of upper airway structures may occur beyond classical gastrointestinal manifestations [28].

A further limitation across the literature is the lack of standardized definitions of ENT involvement. Some studies relied primarily on patient-reported symptoms, whereas others used objective diagnostic tools such as audiometry, endoscopic examination, imaging studies, cytological analysis, or histopathological confirmation. This heterogeneity in diagnostic criteria likely contributes to variability in reported prevalence and currently limits the feasibility of quantitative meta-analysis.

### 4.2. Ear Manifestations: Sensorineural Hearing Loss as a Recurring Finding

The most frequently reported ear-related manifestation in this review was SNHL, appearing in both UC and CD populations. Evidence came from both observational cohorts and case reports. The largest dataset among the included studies identified 32 IBD patients presenting with hearing loss over a 17-year period [10]. Their findings suggested that hearing loss typically develops after IBD onset, often within the first decade of disease, and may occur suddenly in a subset of patients. Importantly, audiometric patterns varied, implying that SNHL in IBD is not a single phenotype but may reflect multiple underlying mechanisms.

Pediatric evidence adds further nuance. It has been demonstrated that pediatric IBD patients may exhibit early high-frequency hearing impairment detectable only through extended audiologic testing, suggesting that SNHL may begin subclinically and progress before symptoms arise [11]. This supports the concept that inflammation-related cochlear damage may develop gradually, with early effects in higher frequencies due to the vulnerability of basal cochlear hair cells.

Conversely, the evidence is not uniform. No statistically significant differences in SNHL were found when studying UC patients in remission compared with controls [13]. This discrepancy may be explained by disease phase, differences in sample size, and the possibility that SNHL affects only a subset of IBD patients.

Additional studies have reported associations between IBD and sensorineural hearing impairment, further supporting the concept of immune-mediated inner ear involvement [47,48]. Case-based evidence also supports this mechanism. For example, symptomatic SNHL has been reported in a patient with UC [49].

This association is also supported by the broader otolaryngology literature. For example, a study documented SNHL in a significant proportion of IBD patients and proposed immune-mediated inner ear injury as a possible cause [50]. Collectively, available evidence indicates that SNHL may represent an underrecognized yet clinically meaningful manifestation of IBD.

### 4.3. Nasal Manifestations: Inflammatory Lesions, Abscess Mimics, and Destructive Complications

Nasal involvement emerged as one of the most clinically striking ENT domains in IBD, particularly due to destructive inflammatory lesions that may mimic infectious processes. A case of nasal pyoderma gangrenosum mimicking a septal abscess in UC has been reported [19]. Despite antibiotic therapy and drainage, the lesion progressed until systemic corticosteroids were administered, producing rapid improvement. However, the patient subsequently developed a saddle-nose deformity, illustrating the severe structural consequences that can result from a delayed diagnosis.

Similarly, an aseptic nasal septal abscess in PR3-ANCA-positive UC with histological evidence of neutrophilic mucositis and phlebitis has been reported [21]. This case required immunosuppressive therapy, highlighting that ENT inflammatory lesions in IBD may overlap with systemic vasculitic disorders. Additional nasal inflammatory pathology included mucosal inflammatory lesions associated with CD and a rare case of nasal mucosa pyoderma vegetans associated with UC, further supporting the role of neutrophilic inflammatory processes in mucosal tissues [15].

Subclinical sinonasal inflammation has also been reported. Increased lymphocytes in nasal cytology have been found to correlate with the degree of intestinal inflammation in UC [23]. In addition, CRS has been reported in association with IBD symptoms [24]. Emerging evidence also suggests a potential bidirectional relationship between CRS and IBD, possibly mediated by shared inflammatory and immune pathways [51].

### 4.4. Laryngeal and Airway Involvement

Laryngeal involvement in IBD appears to be rare but clinically significant, primarily due to the risk of airway compromise. Pediatric UC patients presenting with severe tracheitis have been reported to respond rapidly to systemic corticosteroid therapy [26]. Granulomatous supraglottic edema causing airway obstruction has also been described in a pediatric patient with suspected CD, requiring intensive airway management [27].

Additional inflammatory lesions of the supraglottic region have also been reported, including refractory aphthous lesions involving the epiglottis and supraglottic area in CD [28], further demonstrating that IBD may affect upper airway mucosa beyond the gastrointestinal tract [52].

Voice-related manifestations have also been reported in IBD. Inflammatory involvement of the laryngeal mucosa may lead to symptoms such as dysphonia or voice changes in affected patients [34].

### 4.5. Malignancy Risk and ENT Outcomes

The potential association between IBD and head and neck malignancy is clinically relevant in the context of chronic inflammation and long-term immunosuppressive therapy. Van de Ven et al. [35] reported risk factors for laryngeal carcinoma in patients with IBD, including male sex, tobacco exposure, and certain CD phenotypes. Importantly, survival outcomes appear comparable to those observed in the general population, suggesting that traditional risk factors may play a more significant role than immunosuppressive therapy alone.

### 4.6. Pathophysiological Considerations

Several mechanistic pathways may explain ENT involvement in IBD. Immune dysregulation appears to be central in IBD and may contribute to systemic inflammatory processes affecting extraintestinal organs [53], particularly in manifestations such as SNHL and neutrophilic mucosal lesions. Autoimmune processes affecting inner ear structures may parallel other extraintestinal inflammatory conditions associated with IBD.

Neutrophilic dermatoses such as pyoderma gangrenosum and pyoderma vegetans represent another potential mechanism for destructive nasal disease. These disorders are strongly associated with systemic inflammatory diseases and typically respond well to systemic immunosuppression.

Finally, systemic inflammatory activation may contribute to thrombotic or infectious ENT complications, particularly in immunosuppressed patients.

### 4.7. Clinical Implications

The clinical implications of these findings are significant. Although ENT manifestations remain relatively uncommon, clinicians managing IBD should maintain awareness of potential otologic, sinonasal, and airway complications. Early referral to otolaryngology may be appropriate when patients present with unexplained hearing loss, persistent sinonasal inflammation, or airway symptoms.

Previous literature has emphasized that extraintestinal manifestations affecting head and neck structures may be underrecognized in routine clinical practice [32,42,43]. Greater awareness among gastroenterologists and otolaryngologists may therefore facilitate earlier diagnosis and multidisciplinary management. The key features of the included studies including study design, patient population, ENT findings, diagnostic methods, and outcomes, are summarized in Table 1.

### 4.8. Limitations and Future Directions

Several limitations of the available evidence must be acknowledged. The majority of included studies were case reports or small observational series, limiting the ability to estimate prevalence or establish causal relationships. Publication bias may also influence the available literature, as unusual or severe cases are more likely to be reported. Additionally, a formal risk-of-bias assessment was not performed because most included studies consisted of case reports and small observational series, for which conventional quality assessment tools are not easily applicable. Due to the substantial heterogeneity in study design, patient populations, and reported outcomes, quantitative meta-analysis was not considered appropriate.

Importantly, the therapeutic approaches described across the included studies should not be interpreted as standardized clinical guidelines. In many cases, management strategies were individualized based on specific patient characteristics, disease severity, and clinical context. Therefore, caution is required when extrapolating these findings to broader IBD populations.

Future research should prioritize well-designed multicenter prospective studies incorporating standardized ENT assessment protocols. In addition, the integration of biomarkers of intestinal permeability—such as fecal calprotectin, lactulose–mannitol testing, and lipopolysaccharide-related assays—may help to better define the mechanistic relationship between intestinal inflammation and ENT manifestations.

## 5. Conclusions

This systematic review demonstrates that ENT manifestations in IBD represent a heterogeneous and clinically significant group of extraintestinal complications. Although relatively uncommon, ENT involvement may cause substantial functional impairment and morbidity, ranging from SNHL and chronic sinonasal inflammation to destructive nasal lesions and severe airway disease. The most frequently reported manifestation was SNHL, identified across multiple study designs, including retrospective cohorts, pediatric case–control studies, and case reports, suggesting that inner ear involvement may occur in both UC and CD and may develop years after gastrointestinal disease onset. Evidence indicates variable responsiveness to systemic corticosteroid therapy, supporting an immune-mediated mechanism in selected cases.

Nasal involvement included pyoderma gangrenosum, pyoderma vegetans, and aseptic septal abscess, often mimicking infectious processes. Importantly, delayed diagnosis may lead to irreversible complications such as septal perforation or saddle-nose deformity. Laryngeal and airway manifestations, although rare, may be rapidly progressive and life-threatening, particularly in pediatric patients, reinforcing the need for prompt recognition and early immunosuppressive treatment when inflammatory airway disease is suspected. Subclinical nasal inflammation and chronic rhinosinusitis associations further suggest shared inflammatory pathways between the upper airway mucosa and intestinal immune dysregulation.

The available evidence is limited. Most studies are case reports or small observational series, highlighting the need for larger prospective studies and standardized diagnostic criteria. Early recognition of ENT involvement in patients with IBD may facilitate timely multidisciplinary evaluation and prevent irreversible complications.

## Figures and Tables

**Table 1:** Characteristics of included studies reporting ENT manifestations in inflammatory bowel disease.

Study	Design	Population	ENT Manifestation(s)	Diagnostic Methods	Main Findings	Type of Association with IBD
Birda et al., 2022 [17]	Case report	UC patient	SNHL	Audiologic evaluation	Hearing loss associated with ulcerative colitis	Direct (IBD-related)
Casella et al., 2015 [49]	Case report	UC patients	Symptomatic SNHL	Audiologic testing	Clinical association between UC and hearing impairment	Direct (IBD-related)
Colevas et al., 2020 [27]	Case report	Pediatric patient with suspected CD	Granulomatous supraglottic edema	Laryngoscopy, imaging	Severe airway obstruction requiring tracheostomy	Direct (IBD-related)
Dhillon et al., 2022 [24]	Pilot prospective prevalence study	92 CRS patients screened	CRS associated with IBD	Endoscopy, CT, QoL questionnaires	CRS patients showed increased prevalence of IBD symptoms	Association suggested (bidirectional/epidemiologic)
Hori et al., 2020 [28]	Case report	CD patient	Aphthous lesions of epiglottis	Laryngoscopy	Inflammatory supraglottic lesions related to CD	Direct (IBD-related)
Ishikawa et al., 2022 [21]	Case report	PR3-ANCA positive UC	Aseptic nasal septal abscess	CT, histology, serology	Responded to immunosuppressive therapy	Immune-mediated/IBD-related
Kinoshita et al., 2024 [10]	Retrospective study	32 IBD patients	SNHL	Audiometry, imaging	Hearing loss occurred after IBD onset in most cases	Direct (IBD-related)
Marvisi et al., 2019 [23]	Observational study	UC patients	Subclinical nasal inflammation	Nasal cytology	Nasal lymphocytosis correlated with intestinal activity	Subclinical association (IBD activity-related)
Murayama et al., 2026 [36]	Case report	UC patient	Sterile nasal septal abscess	Clinical exam, immunologic testing	Improved with corticosteroids and IVIG	Medication/immune-related (unclear)
Nunes et al., 2016 [26]	Case series	Pediatric UC patients	Tracheitis	Laryngofibroscopy	Rapid improvement with systemic steroids	Direct (IBD-related)
Polat et al., 2020 [11]	Case–control	Children with IBD	High-frequency SNHL	High-frequency audiometry	Early cochlear involvement suggested	Direct (IBD-related)
Sagit et al., 2016 [13]	Prospective study	UC patients in remission	SNHL	Pure tone audiometry	No significant difference vs. controls	No association (remission cohort)
Tirelli et al., 2015 [14]	Case report	CD patient	Sudden SNHL	Audiologic evaluation	Possible autoimmune inner ear involvement	Immune-mediated (suspected IBD-related)
Tomioka et al., 2018 [19]	Case report	UC patient	Nasal pyoderma gangrenosum	ENT exam, CT	Led to saddle-nose deformity despite treatment	Direct (IBD-related)
Unić et al., 2018 [31]	Case report	Adolescent with UC	Lemierre syndrome	Imaging, microbiology	Severe infectious complication	Infectious complication (secondary to IBD/immunosuppression)
Vahedi et al., 2015 [15]	Case report	UC patient	Nasal mucosa pyoderma vegetans	ENT exam, histopathology	Rare neutrophilic lesion responsive to corticosteroids	Direct (IBD-related)
Vavricka et al., 2015 [18]	Multicenter case series	UC and CD	Cogan’s syndrome	Clinical assessment	Audiovestibular autoimmune syndrome associated with IBD	Direct (IBD-related)
van de Ven et al., 2020 [35]	Retrospective study	IBD patients	Laryngeal carcinoma	Pathology database	Risk factors include male sex and smoking	Indirect (risk factors predominant)
Wengrower et al., 2016 [12]	Observational study	IBD patients	Hearing loss	Audiologic evaluation	Characterized hearing patterns in IBD	Association suggested
Yazici et al., 2015 [16]	Case report	UC patient	SNHL with neurologic symptoms	Audiologic and neurologic evaluation	Suggested immune-mediated mechanism	Immune-mediated (IBD-related)
Yin et al., 2023 [29]	Case report	CD patient	Laryngeal inflammatory lesion	Laryngoscopy	Rare laryngeal manifestation of CD	Direct (IBD-related)
Yu et al., 2020 [20]	Case report	UC patient	Nasal mucosa pyoderma vegetans	ENT exam, biopsy	Complete response to prednisone	Direct (IBD-related)
Zaseeva et al., 2025 [22]	Case report	CD patient	Intranasal inflammatory lesion	Nasal endoscopy, imaging, biopsy	CD presenting as intranasal space-occupying lesion	Direct (IBD-related)

Abbreviations: CD, Crohn’s disease; CT, computed tomography; ENT, ear, nose and throat; IBD, inflammatory bowel disease; SNHL, sensorineural hearing loss; UC, ulcerative colitis; PR3-ANCA, proteinase 3 anti-neutrophil cytoplasmic antibodies.

## Data Availability

Data sharing is not applicable as no new data were generated in this study.

## Supplementary Materials

The following supporting information can be downloaded at: https://www.mdpi.com/article/10.3390/medicina62050943/s1, Supplementary Material S1: Detailed search strategy for PubMed and Scopus databases; Supplementary Material S2: Table S1: Extended characteristics of included studies-PRISMA Checklist. Ref. [54] is cited in Supplementary Materials S2.
