# Supplementary material for: Ear, Nose, and Throat Manifestations in Inflammatory Bowel Diseases: A Systematic Review of the Clinical Spectrum
Source: Medicina (Kaunas). 2026 May 12;62(5):943. doi: 10.3390/medicina62050943 (PMC13208139; doi:10.3390/medicina62050943)
Supplement: Supplementary file 1 [file medicina-62-00943-s001.zip › Supplementary Material S1-Search Strategy.pdf]

Supplementary Material

Search Strategy

PubMed Search Strategy

(("Inflammatory Bowel Diseases"[Mesh] OR "inflammatory bowel disease" OR "ulcerative colitis" OR "Crohn's disease")

AND

("Otorhinolaryngologic Diseases"[Mesh] OR "ear" OR "nose" OR "throat" OR "ENT" OR "hearing loss" OR "sensorineural hearing loss" OR "sinusitis" OR "rhinosinusitis" OR "nasal" OR "laryngeal" OR "airway" OR "voice"))

Filters applied: English language; human studies; publication dates from January 1, 2015, to January 25, 2026.

Scopus Search Strategy

(TITLE-ABS-KEY ("inflammatory bowel disease" OR "ulcerative colitis" OR "Crohn's disease")

AND

TITLE-ABS-KEY ("ear" OR "nose" OR "throat" OR "ENT" OR "hearing loss" OR "sensorineural hearing loss" OR "sinusitis" OR "rhinosinusitis" OR "nasal" OR "laryngeal" OR "airway" OR "voice"))

Filters applied: English language; document type limited to articles; publication years 2015–2026.
